# Low Prevalence of *Schistosoma mekongi* Infection and High Prevalence of Other Helminth Infections among Domestic Animals in Southern Lao People’s Democratic Republic

**DOI:** 10.3390/tropicalmed8070372

**Published:** 2023-07-18

**Authors:** Somphou Sayasone, Phonepadith Khattignavong, Sengdeuane Keomalaphet, Phoyphaylinh Prasayasith, Pheovaly Soundala, Sonesimmaly Sannikone, Takashi Kumagai, Souk Phomhaksa, Phouth Inthavong, Emilie Louise Akiko Matsumoto-Takahashi, Bouasy Hongvanthong, Paul T. Brey, Shigeyuki Kano, Moritoshi Iwagami

**Affiliations:** 1 Lao Tropical and Public Health Institute, Ministry of Health, Vientiane Capital 0103, Laos; somphou.sayasone@yahoo.com; 2 Institut Pasteur du Laos, Ministry of Health, Vientiane Capital P.O. Box 3560, Laos; phonepadithk@gmail.com (P.K.); ksengdeuan@yahoo.com (S.K.); 2noknoy.phoy@gmail.com (P.P.); pheovaly@gmail.com (P.S.); sannikonedad@gmail.com (S.S.); p.brey@pasteur.la (P.T.B.); kano@ri.ncgm.go.jp (S.K.); 3 Department of Parasitology and Tropical Medicine, Tokyo Medical and Dental University, 1-5-45 Yushima, Bunkyo, Tokyo 113-8510, Japan; tkuma.vip@tmd.ac.jp; 4 National Animal Health Laboratory, Department of Livestock and Fishery, Ministry of Agriculture and Forestry, Vientiane Capital P.O. Box 6644, Laosdrphouth@yahoo.com (P.I.); 5 Graduate School of Public Health, St. Luke’s International University, 3-6-2-5F Tsukiji, Chuo, Tokyo 104-0045, Japan; etakahashi@slcn.ac.jp; 6 Department of Tropical Medicine and Malaria, Research Institute, National Center for Global Health and Medicine, 1-21-1 Toyama, Shinjuku, Tokyo 162-8655, Japan; 7 Center of Malariology, Parasitology and Entomology, Ministry of Health, Vientiane Capital P.O. Box 0100, Laos; cmpelao@gmail.com

**Keywords:** *Schistosoma mekongi*, LAMP, reservoir animal, domestic animal, Lao PDR, Mekong River

## Abstract

The prevalence of *Schistosoma mekongi* in humans in the Lao People’s Democratic Republic (Lao PDR) has been relatively well monitored and has decreased due to effective interventions such as preventative chemotherapy with mass drug administration of praziquantel and community awareness programs. However, the prevalence among potential domestic reservoir animals remains broadly unclear, except for a few villages in the endemic area. Therefore, we conducted *S. mekongi* surveys for the domestic animals that had contact with Mekong River water. We conducted a cross-sectional study of the domestic animals in the seven sentinel villages in the Khong and Mounlapamok Districts of Champasak Province in southern Lao PDR in 2018 by random sampling with a statistically reliable sample size. Stool samples of the five predominant domestic animal species, cattle (n = 160), pig (n = 154), buffalo (n = 149), dog (n = 143), and goat (n = 85), were collected and examined using parasitological FECT method and the LAMP technique. The microscopic analysis did not detect any eggs of *S. mekongi* in the stool samples of any animal species. However, *S. mekongi* DNA was detected by the LAMP test in dog stool samples (0.7%; 1/143). On the other hand, the prevalence of other helminths was quite high and heterogeneous among animal species and sentinel sites by the microscopic analysis. These findings suggested that an intervention for *S. mekongi* infection should focus solely on human populations. However, periodic surveillance for *S. mekongi* infection among dogs should be conducted to monitor a possible resurgence of *S. mekongi* infection in the domestic animal population.

## 1. Introduction

Schistosomiasis is a parasitic disease caused by blood flukes (trematode) of the genus *Schistosoma*. Schistosomiasis is one of the neglected tropical diseases (NTDs) targeted for elimination by the World Health Organization (WHO) [1]. According to the WHO, at least 251.4 million people worldwide require preventive chemotherapy (or mass drug administration: MDA) [2].

*Schistosoma mekongi* is endemic along the Mekong River basin in Champasak Province, the southern part of the Lao People’s Democratic Republic (Lao PDR) and Kratié Province, the northern part of Cambodia. In the Lao PDR, two Districts: Khong (152 villages: 86,095 people) and Mounlapamok (50 villages: 37,063 people), are the endemic areas of *S. mekongi* [3,4]. Significant progress has been made in the past decades to reduce the prevalence of schistosomiasis mekongi through preventive chemotherapy using praziquantel and community awareness programs or health education [5]. According to the results of the recent reports on schistosomiasis mekongi, the prevalence of human schistosomiasis ranged from 0.0% to 5.0% without heavy infection in sentinel sites and endemic communities using Kato–Katz method for parasite egg detection in stool samples [5,6]. Although the prevalence of human schistosomiasis has been relatively well monitored and gradually declining over the past decades in the Lao PDR, the prevalence among potential domestic reservoir animals in the endemic areas has not been well studied. A few studies with limited sample size showed that 14.7% (10/68) of dogs in Donkhone and Donsom in 2011–2012 [7] and 12.2% (12/98) of pigs in Had Xay Khoun village in 1999 were infected with *S. mekongi* [8]. Previous studies report that cats, buffaloes, and cattle were not infected with *S. mekongi* in Donkhone and Donsom. However, it is important to note that these studies used solely the traditional diagnostic method, e.g., microscopy. Therefore, it is possible that light-intensity infections could be missed due to the relatively low sensitivity of the tests. 

In 2017, WHO adopted a new strategy that accelerates the elimination of Asian schistosomiasis in the Western Pacific Region, i.e., transmission interruption by 2025 and verifying elimination by 2030 [1]. One of the criteria of transmission interruption is “no new case of animal infection.” Toward this goal, we conducted a cross-sectional study to determine the infection status of *S. mekongi* among the potential domestic reservoir animals with adequate sample size and high-sensitivity diagnostic methods in the endemic villages in Khong and Mounlapamok Districts, Champasak Province, southern Lao PDR. The prevalence of other helminth infections among domestic animals was also shown in the present study.

## 2. Methods

### 2.1. Study Area and Design

This study was conducted along the Mekong River basin in the Khong and Mounlapamok Districts in Champasak Province in the southern part of the Lao PDR in 2018. These two districts are *S. mekongi* endemic areas. Five villages in Khong District and two villages in Mounlapamok District were selected for the study sites. These villages were selected by the Lao Ministry of Health as sentinel sites for monitoring the prevalence of schistosomiasis in 2017. Five domestic animal species: cattle, buffalos, dogs, pigs, and goats, were investigated in this study.

### 2.2. Sample Size Calculation

Assuming 10% of prevalence of schistosomiasis in animals [7] using 5% precision with a 95% confidence interval, the sample size is 139 for each animal species. Thus, 140 animals per species were enrolled in this study (20 animals per village × 7 villages = 140 animals per species). This assumption of the 10% of prevalence was taken from the highest prevalence of human schistosomiasis in this endemic area in 2016.

### 2.3. Sample Collection from the Field

**Figure 1:**
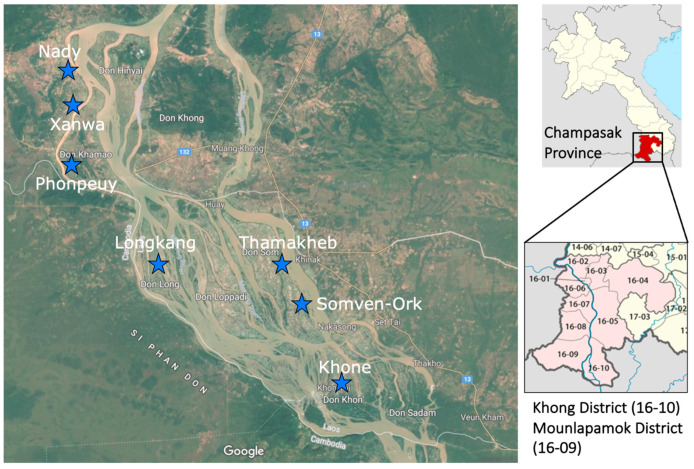
Map of study villages: star marks represent sentinel site villages in Khong and Mounlapamok Districts, Champasack Province, southern part of Lao PDR (Source: Google Maps and Wikipedia).

Three field surveys were conducted in the seven sentinel sites (Khone, Longkang, Thamakheb, Somven-Ork, and Phonpheuy) in Khong District and two sentinel sites (Xanwa and Nady) in Mounlapamok District, Champasak Province, Lao PDR (Figure 1). The first survey was conducted in Longkang, Thamakheb, and Somvenok villages between 6 and 23 May 2018. The second survey was conducted in Khone and Phonpheuy villages between 1 and 15 July 2018. The third survey was conducted in Xanwa and Nady villages between 22 July and 2 August 2018. In each field study, a meeting with villagers and village authorities was conducted to explain the aim and field activities. Prior to sample collection, a list of domestic animals in the study villages was created with the assistance of the villagers and village chief. For each animal species, the number of animals per household was counted. Based on the list, 20 individual animals per species were randomly selected per study village. In a village where the number of animals was 20 or less per species, all animals were enrolled in the study. Approximately 3.0 g of stool samples from each study animal were collected. Exactly 2.0 g of collected samples were preserved in 10% formalin and transported to the Lao Tropical and Public Health Institute, Vientiane Capital, Lao PDR, for parasitological analysis to detect the eggs of helminth parasites. Another 0.5 g of samples were preserved in 70% ethanol for loop-mediated isothermal amplification (LAMP) analysis to detect *S. mekongi* DNA.

### 2.4. Sample Processing

#### 2.4.1. FECT Analysis for Helminth Infections

The stool samples preserved in 10% formalin were further processed at the Lao Tropical and Public Health Institute using the formalin-ethyl acetate concentration technique (FECT) as described previously [9,10]. The prepared slides were placed under a light microscope and read by experienced microscopists. All detected helminth parasite eggs were identified and recorded separately by species. About 10% of the reading slides were re-examined by a senior laboratory technician. Any discrepancy in findings was discussed among microscopists to conclude the consensus findings.

#### 2.4.2. LAMP Technique for the Detection of *Schistosoma mekongi* DNA

The LAMP test for detecting *S. mekongi* DNA was performed in a laboratory at Institut Pasteur du Laos. Primers for the LAMP test were designed based on the internal transcribed spacer 1 (ITS1) region in the ribosomal RNA gene of *S. mekongi* [11]. Positive control DNA for the *S. mekongi* LAMP was extracted from *S. mekongi* adult worms collected from a previous study. *S. mekongi* DNA was extracted by alkali-boil method from the stool samples preserved in 70% ethanol [11]. The extracted DNA was applied to a LAMP reaction tube containing *S. mekongi*-specific primers with an enzyme (Loopamp^TM^ DNA Amplification Kit, Eiken Chemical, Co., Ltd., Tokyo, Japan) and Fluorescent Detection Reagent (Eiken Chemical, Co., Ltd., Tokyo, Japan). The reaction tubes were incubated at 65 °C for 60 min and then incubated at 80 °C for 5 min for the inactivation of DNA amplification enzyme by Loopamp^TM^ LF-160 Incubator (Eiken Chemical, Co., Ltd., Japan). The result of the LAMP test was evaluated using a detector unit (UV light) of the LF-160 Incubator. When *S. mekongi* DNA is present in the stool samples, the DNA is amplified by the LAMP reaction and can then be detected by a color change (transparent to green) under UV light as well as by a change in turbidity (transparent to white) by the naked eye.

### 2.5. Statistical Analysis

Statistical Package for Social Sciences (SPSS) version 27 (IBM Corp., Armonk, NY, USA) was used to analyze the data. Pearson’s chi-square test was used to analyze the prevalence of helminth infections by animal species or villages. A *p*-value less than 0.05 is considered statistically significant. 

## 3. Results

### 3.1. Demography of the Study Population

A total of 699 domestic animals in seven sentinel sites in two endemic districts (Khong and Mounlapamok), Champasak Province, southern Lao PDR, were enrolled in the study. Of these 699 animals, 691 (98.9%) had enough stool samples for both LAMP and FECT analyses, which included 160 cattle, 154 pigs, 149 buffaloes, 143 dogs, and 85 goats. Nady (124 animals) was the village with the highest number of study animals, followed by Phonpeuy (123 animals), Khone (107 animals), Xanwa (101 animals), Thamakheb (88 animals), Longkang (77 animals), and Somven-Ork (71 animals), respectively (Table 1). The number of animals differs among villages (Pearson’s chi-square test). Buffaloes, cattle, dogs, and pigs were present in all villages, but goats were absent in Longkang and Somven-Ork (*p* < 0.001).

### 3.2. Microscopic Findings Using the FECT

From the 691 study animals, the FECT analysis detected helminth infections in 72.4% of the study animals (500/691) (Table 2) and sentinel sites (Table 3). Domestic animals in Nady village had the highest rate of helminth infections (79.8%), followed by animals in Phonpeuy village (79.7%), Thamakheb village (73.9%), Khone village (73.8%), Xanwa village (70.3%), in Somven-Ork village (62.0%), and in Longkang village (57.1%), respectively (Table 3). The prevalence of all helminth infections was significantly different among the sentinel sites (*p* < 0.05) (Table 3). Prevalence of *Ascaris* spp. infection was also different among the sentinel sites (*p* < 0.05). In the study areas, *Haplorchis taichui* and other minute intestinal fluke infections are common in humans and animals. Their eggs morphologically resemble those of *Opisthorchis viverrini*. Thus, we described *O. viverrini*-like eggs in Table 2, Table 3 and Table 4.

Table 4 displays the helminth infections detected in the FECT analysis by animal species. Stool analysis of the study animals did not detect any *S. mekongi* eggs in all preserved samples. *Fasciola* spp. infection in cattle and buffalo was detected in 65.0% and 60.4%, respectively. Hookworm and *Diphylobothrium latum* infections in dogs were 62.5% and 46.5%, respectively. *Trichuris suis*, *Ancylostoma* spp. (hookworm), and *Ascaris* spp. infections in pigs were 19.5%, 40.9%, and 39.6%, respectively. *Trichuris suis*, hookworm, and *Fasciola* spp. infection rates in goats were 28.2%, 67.1%, and 32.9%, respectively. The prevalence of each helminth was statistically heterogeneous (*p* < 0.05) except for the prevalence of *Hymenolepis nana*.

*Fasciola* spp. eggs had the highest rate of helminth infections among animals in Khone (42.1%), Xanwa (32.7%), Somven-Ork (28.2%), and Longkang (23.4%) villages, while hookworm had the highest rates of helminth infections for animals in Nady (40.3%), Phonpeuy (36.6%), and Thamakheb (31.8%) villages (Table 4).

### 3.3. Findings from the Molecular Detection Using the LAMP

From 691 study animals, a stool sample from a dog (0.7%, 1/143) and a stool sample from a pig (0.6%, 1/154) were *S. mekongi*-DNA-positive using the LAMP method in Phonpeuy village. The validation of the positivity was performed in three independent tests, including DNA extraction. Only one dog sample was positive by the three independent LAMP tests, and it was counted as a confirmed positive (Table 5). For the pig sample, the positive result was obtained on only one test, along with two negative results. Thus, the pig sample was considered a suspected positiv, rather than a confirmed positive. The age of the *S. mekongi*-positive dog was 4 years old, while the pig was only 3 months old. All the other stool samples were *S. mekongi*-DNA-negative by the LAMP test.

## 4. Discussions

In the present study, *S. mekongi* DNA was detected from only one dog and one pig stool sample by the LAMP test. These dog and pig stool samples were considered confirmed and suspected positive, respectively, due to the varied results for the pig sample by the LAMP test. Microscopic examination using the FECT method showed negative results with the same stool samples. These different results suggested that the LAMP test is more sensitive than the FECT. In our previous study, the sensitivity and specificity of microscopic examination using the Kato–Katz method for detecting *S. mekongi* eggs in the stool samples were evaluated in comparison to the LAMP test as the gold standard, and they were 12.5% and 100.0%, respectively [11]. The detection limit of the LAMP test was 1 pg of *S. mekongi* DNA/reaction, whereas neither *S. japonicum* nor *S. mansoni* DNA were detected by this *S. mekongi* LAMP test (no cross-reaction). All stool samples from other domestic animals (cattle, buffalo, and goats) were negative for *S. mekongi* by both the LAMP test and the FECT.

Microscopic examination by the FECT detected several helminth eggs except *S. mekongi* egg in this study. The prevalence of each helminth species was heterogeneous among the animals and sentinel sites. For example, *Fasciola* ssp. was highly prevalent in buffalo (60.4%) and cattle (65.0%) compared to goats (32.9%) and pigs (0.6%) (*p* < 0.05). The reason for the low rate of *Fasciola* ssp. infection in pigs probably lies in the rearing environment. Most pigs were tied up or kept in pens during our survey so that they had a low risk of eating *Fasciola* ssp.-contaminated freshwater plants. The heterogeneity of the prevalence of helminths among villages was also observed. For example, the animals in Nady were highly contaminated by several helminths, such as *D. latum*, *Fasciola* ssp. and hookworm. However, the cause of the high infection rates of the helminths in Nady was unknown in this study.

Our previous study between October 2011 and August 2012 showed that 14.7% of dogs (10/68) were positive for *S. mekongi* eggs using the FECT method in two islands (Donkhone, 15.9%, and Donsom, 12.5%). The prevalence of schistosomiasis among humans was as high as 23.6% (112/475) in Donkhone (Khone island) and 21.0% (109/519) in Donsom (Som Island), with heavy-intensity infections of 1.8% (2/112) and 5.5% (6/109), respectively [7]. It is important to note that the settings of our present study geographically overlap with the previous study conducted in 2011 and 2012. Khone village is in Donkhone, and Thamakheb and Somven-Ork villages are in Donsom. The most recent study showed a much lower prevalence of schistosomiasis among humans in Khone, Thamakheb, and Somven-Ork at 7.3%, 6.0%, and 4.5%, respectively, without a heavy-intensity infection [5]. Several efforts have been made by the Lao government and development partners to reduce the prevalence of schistosomiasis among humans in endemic areas. The key components of the intervention were the annual MDA with praziquantel, health education, installation of latrines and water-pump systems through the Community-Led initiatives to eliminate Schistosomiasis with Water Supply, Sanitation and Hygiene (CL-SWASH) in the endemic areas. Although pregnant and lactating women were excluded from the annual MDA, the prevalence of schistosomiasis gradually decreased, and a case of heavy-intensity infection disappeared from the sentinel sites. Children were almost free from schistosomiasis in the study areas. In addition, our previous snail host (*Neotricula aperta*) survey using the LAMP method demonstrated that the *S. mekongi* infection rate of the snail was 0.26% in 2016, 0.08% in 2017, and 0.03% in 2018 in Donkhone [11]. The low prevalence of *S. mekongi* infection in the animals observed in this study may be associated with the significant reduction of schistosomiasis in these endemic communities (human populations) and the significant reduction of the infection rate of the snail in Donkhone, even though no intervention was performed for the animals in the study areas.

In addition, 88.2% of dogs (127/144) in the present study were less than 3 years old (average 2.2 years old), whereas the *S. mekongi*-positive dog was 4 years old. Although the age of the dogs may not be accurate because there is no official age documentation, this result suggests that *S. mekongi* transmission among dogs has either not occurred or has been limited since 2015 in the study areas.

In our previous study conducted in 2011–2012, the stool samples of cats (n = 64), pigs (n = 105), and buffaloes (n = 94) were also examined using the FECT method and were all negative for *S. mekongi* eggs. However, another study conducted in Hadxaykhoun village, Khong District, in 1999 found a positive rate for *S. mekongi* eggs in 12.2% (12/98) of pigs [8]. Strandgaard et al. suspected that the most likely route of pig infection was ingestion of cercariae-infested drinking water that the owners brought directly from the Mekong River to feed their pigs daily. At that time (1999), most pigs were normally tied up or kept in pens, but occasionally they were allowed to roam freely. During our study, we observed a similar manner of keeping pigs in 1999 in Khong District. However, the extensive intervention conducted over the past decades has reduced the prevalence of *S. mekongi* in the endemic areas to a very low level, with no severe cases observed in the community [5]. In addition, we observed that most of the households in the endemic communities pumped the water from the Mekong River and stored it in a tank or well for hours before using it. Previous studies suggested that *Schistosoma* cercariae can remain infective in freshwater for one to three days [12], depending on the water temperature [13,14]. If the owner of the pigs gave the storage water (stored for more than three days) to their pigs, the risk of pig infection with *S. mekongi* would be lower compared with previous practices. Moreover, the age of the pigs in this study was relatively young, with an average of 5 months (ranging from 1 month to 2 years old). In fact, the study team did not see any older pigs (>2 years old) in this study. This relatively high turnover of the pig population would decrease the chance of *S. mekongi* infection in the study areas.

**Figure 2:**
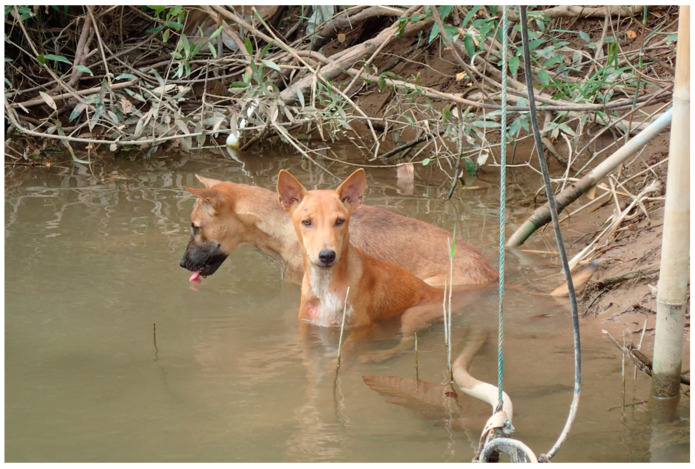
Two dogs stay in the Mekong River in an endemic village, Khone Island (Donkhone), Khong District, Champasak Province in 2018 (Photo: M Iwagami).

In the present surveys, we observed that many dogs took baths on the Mekong riverside (Figure 2), while no pigs came to the Mekong River as they were normally kept in pens. Buffaloes like to stay in the water for a long time. Cattle and goats normally come to the Mekong River’s riverside only when they want to drink water. *S. mekongi* is genetically and morphologically close to *S. japonicum*, which has a wide variety of mammal hosts, such as cattle, buffalo, pigs, dogs, rats, and so on [3,15]. In contrast, *S. mekongi* has only two mammalian hosts as natural reservoirs, namely, dogs and pigs in the endemic areas [7,8]. Rats are a mammalian host in a laboratory setting [16], and buffaloes are suspected as a potential reservoir animals [3], but this has yet to be proven. The present study and the accumulated data from previous studies suggest that only dogs and pigs are the natural reservoir animals for *S. mekongi*. Rats were not examined in this study because they are not domestic animals, and it is challenging to collect them. Therefore, it remains unknown whether rats would contribute to the transmission of *S. mekongi* in the endemic areas.

## 5. Conclusions

This study found only one *S. mekongi*-positive case in dogs and one suspicious case in pigs by the LAMP test in the seven sentinel sites in Lao PDR in 2018. No *S. mekongi* infection was observed among cattle, buffaloes, or goats. Therefore, we conclude that humans play a key role in the transmission of *S. mekongi* in the endemic areas of Khong and Mounlapamok Districts, Champasak Province, southern Lao PDR. Therefore, key interventions focusing on humans, such as community-based chemotherapy coupled with health education and improving access to safe water and sanitation, might significantly reduce the *S. mekongi* infection in the endemic areas, leading to its elimination.

## Figures and Tables

**Table 1:** Numbers of animal screening, stratified by species and villages.

	Buffalo (n = 149)	Cattle (n = 160)	Dog (n = 143)	Goat (n = 85)	Pig (n = 154)	Total (n = 691)	*p*-Value *
Khone	25	36	22	18	6	107	<0.001
Longkang	18	22	19	0	18	77	
Naby	21	25	20	33	25	124	
Phoneuy	20	30	24	11	38	123	
Somven-Ork	18	18	20	0	15	71	
Thamakheb	22	3	20	19	24	88	
Xamwa	25	26	18	4	28	101	

* Pearson’s chi-square test.

**Table 2:** Helminth infections among study animals detected by FECT.

	Buffalo (n = 149)		Cattle (n = 160)		Dog (n = 143)		Goat (n = 85)		Pig (n = 154)		Total (N = 691)		*p*-Value *
	n	%	n	%	n	%	n	%	n	%	n	%	
All helminths	97	65.1	112	70.0	122	85.3	68	80.0	101	65.6	500	72.4	<0.001
*Ascaris* ssp.	0	0.0	1	0.6	2	1.4	3	3.5	61	39.6	67	9.7	<0.001
*Diphyllobothrium latum*	0	0.0	0	0.0	67	46.9	0	0.0	0	0.0	67	9.7	<0.001
*Fasciola* ssp.	90	60.4	104	65.0	0	0.0	28	32.9	1	0.6	223	32.3	<0.001
*Hymenolepis nana*	0	0.0	0	0.0	1	0.7	0	0.0	0	0.0	1	0.1	0.428
Hookworm	0	0.0	6	3.8	89	62.2	57	67.1	63	40.9	215	31.1	<0.001
*Opisthorchis viverrini*-like egg	0	0.0	0	0.0	4	2.8	0	0.0	0	0.0	4	0.6	<0.004
*Trichuris suis*	2	1.3	1	0.6	7	4.9	24	28.2	30	19.5	64	9.3	<0.001

* Pearson’s chi-square test.

**Table 3:** Helminth infections from seven villages (sentinel sites) detected by FECT.

Helminths	Village																*p*-Value *
	Khone (n = 107)		Longkang (n = 77)		Naby (n = 124)		Phonpeuy (n = 123)		Spmven-Ork (n = 71)		Thamakheb (n = 88)		Xanwa (n = 101)		Total (N = 691)		
	n	%	n	%	n	%	n	%	n	%	n	%	n	%	n	%	
All helminths	79	73.8	44	57.1	99	79.8	98	79.7	44	62.0	65	73.9	71	70.3	500	72.4	<0.003
*Ascaris* ssp.	3	2.8	6	7.8	6	4.8	20	16.3	7	9.9	11	12.5	14	13.9	67	9.7	<0.005
*Diphyllobothrium latum*	12	11.2	4	5.2	14	11.3	11	8.9	6	8.5	6	6.8	8	7.9	61	8.8	0.602
*Fasciola* ssp.	45	42.1	18	23.4	47	37.9	36	29.3	20	28.2	24	27.3	33	32.7	223	32.3	0.078
*Hymenolepis nana*	0	0.0	0	0.0	0	0.0	0	0.0	0	0.0	1	1.1	0	0.0	1	0.1	0.334
Hookworm	30	28.0	16	20.8	50	40.3	45	36.6	18	25.4	28	31.8	28	27.7	215	31.1	0.050
*Opisthorchis viverrini*-like egg	0	0.0	0	0.0	0	0.0	1	0.8	0	0.0	2	2.3	1	1.0	4	0.6	0.320
*Trichuris suis*	13	12.1	2	2.6	13	10.5	11	8.9	2	2.8	10	11.4	13	12.9	64	9.3	0.091

* Pearson’s chi-square test.

**Table 4:** Differences in the number of parasitic infections per study animal and village detected by FECT.

Animals	Parasites	Village																*p*-Value ^1^
		Khone (n = 107)		Longkang (n = 77)		Naby (n = 124)		Phonpeuy (n = 123)		Spmven-Ork (n = 71)		Thamakheb (n = 88)		Xanwa (n = 101)		Total (N = 691)		
		n	%	n	%	n	%	n	%	n	%	n	%	n	%	n	%	
Buffalo (n = 149)	All parasites	17	68.0	4	22.2	18	85.7	18	90.0	10	55.6	14	63.6	16	64.0	97	65.1	<0.001
	*Ascaris* ssp.	0	0	0	0	0	0	0	0	0	0	0	0	0	0	0	0	-
	*Diphyllobothrium latum*	0	0	0	0	0	0	0	0	0	0	0	0	0	0	0	0	-
	*Fasciola* ssp.	17	68.0	3	16.7	17	81.0	14	70.0	9	50.0	14	63.6	16	64.0	90	60.4	0.002
	*Hymenolepis nana*	0	0	0	0	0	0	0	0	0	0	0	0	0	0	0	0	-
	Hookworm	0	0	0	0	0	0	0	0	0	0	0	0	0	0	0	0	-
	*Opisthorchis viverrini*-like egg	0	0	0	0	0	0	0	0	0	0	0	0	0	0	0	0	-
	*Trichuris suis*	0	0	0	0	0	0	0	0	1	5.6	1	4.5	0	0	2	1.3	0.499
Cattles (n = 160)	All parasites	27	75.0	16	72.7	19	76.0	21	70.0	11	61.1	2	66.7	16	61.5	112	70.0	0.867
	*Ascaris* ssp.	1	2.8	0	0	0	0	0	0	0	0	0	0	0	0	1	0.6	0.748
	*Diphyllobothrium latum*	0	0	0	0	0	0	0	0	0	0	0	0	0	0	0	0	-
	*Fasciola* ssp.	25	69.4	15	68.2	18	72.0	19	63.3	11	61.1	2	66.7	14	53.8	104	65.0	0.865
	*Hymenolepis nana*	0	0	0	0	0	0	0	0	0	0	0	0	0	0	0	0	-
	Hookworm	2	5.6	1	4.5	1	4.0	1	3.3	0	0	0	0	1	3.8	6	3.8	0.977
	*Opisthorchis viverrini*-like egg	0	0	0	0	0	0	0	0	0	0	0	0	0	0	0	0	-
	*Trichuris suis*	0	0	0	0	0	0	1	3.3	0	0	0	0	0	0	1	0.6	0.628
Dogs (n = 143)	All parasites	18	81.8	15	78.9	20	100.0	22	91.7	14	70.0	17	85.0	16	88.9	122	85.3	0.175
	*Ascaris* ssp.	0	0	0	0	0	0	1	4.2	1	5.0	0	0	0	0.0	2	1.4	0.594
	*Diphyllobothrium latum*	12	54.5	4	21.1	14	70.0	11	45.8	6	30.0	12	60.0	8	44.4	67	46.9	0.034
	*Fasciola* ssp.	0	0	0	0	0	0	0	0	0	0	0	0	0	0	0	0	-
	*Hymenolepis nana*	0	0	0	0	0	0	0	0	0	0	1	5.0	0	0	1	0.7	0.402
	Hookworm	14	63.6	10	52.6	18	90.0	20	83.3	12	60.0	5	25.0	10	55.6	89	62.2	<0.001
	*Opisthorchis viverrini*-like egg	0	0	0	0	0	0	1	4.2	0	0	2	10.0	1	5.6	4	2.8	0.338
	*Trichuris suis*	2	9.1	1	5.3	0	0	0	0	0	0	1	5.0	3	16.7	7	4.9	0.148
Goats ^2^ (n = 85)	All parasites	15	83.3	-	-	23	69.7	10	90.9	-	-	16	84.2	4	100	68	80.0	0.362
	*Ascaris* ssp.	0	0	-	-	0	0	0	0	-	-	3	15.8	0	0	3	3.5	0.029
	*Diphyllobothrium latum*	0	0	-	-	0	0	0	0	-	-	0	0	0	0	0	0	-
	*Fasciola* ssp.	3	16.7	-	-	11	33.3	3	27.3	-	-	8	42.1	3	75	28	32.9	0.182
	*Hymenolepis nana*	0	0	-	-	0	0	0	0	-	-	0	0	0	0	0	0	-
	Hookworm	13	72.2	-	-	18	54.5	10	90.9	-	-	12	63.2	4	100	57	67.1	0.112
	*Opisthorchis viverrini*-like egg	0	0	-	-	0	0	0	0	-	-	0	0	0	0	0	0	-
	*Trichuris suis*	11	61.1	-	-	6	18.2	0	0	-	-	4	21.1	3	75	24	28.2	<0.001
Pigs (n = 154)	All parasites	2	33.3	9	50.0	19	76.0	27	71.1	9	60.0	16	66.7	19	67.9	101	65.6	0.350
	*Ascaris* ssp.	2	33.3	6	33.3	6	24.0	19	50.0	6	40.0	8	33.3	14	50.0	61	39.6	0.389
	*Diphyllobothrium latum*	0	0	0	0	0	0	0	0	0	0	0	0	0	0	0	0	-
	*Fasciola* ssp.	0	0	0	0	1	4.0	0	0	0	0	0	0	0	0	1	0.6	0.519
	*Hymenolepis nana*	0	0	0	0	0	0	0	0	0	0	0	0	0	0	0	0	-
	Hookworm	1	16.7	5	27.8	13	52.0	14	36.8	6	40.0	11	45.8	13	46.4	63	40.9	0.560
	*Opisthorchis viverrini*-like egg	0	0	0	0	0	0	0	0	0	0	0	0	0	0	0	0	-
	*Trichuris suis*	0	0	1	5.6	7	28.0	10	26.3	1	6.7	4	16.7	7	25.0	30	19.5	0.224

^1^ Pearson’s chi-square test. ^2^ No goats in Longkang and Spmven-Ork village. Percentage (%) of animals in each village.

**Table 5:** Prevalence of *Schistosoma mekongi* in study animals detected by LAMP method.

Animals	No. of Examined	Suspected, n (%)	Positive, n (%)
Dog	143	0	1 (0.7)
Pig	154	1 (0.6)	0
Cattle	160	0	0
Buffalo	149	0	0
Goat	85	0	0

## Data Availability

All data collected from the field and analyzed for this manuscript were available at the Intitut Pasteur du Laos and the Lao Tropical and Public Health Institute. Data will make freely access to interested individuals and institutions upon the official request.
